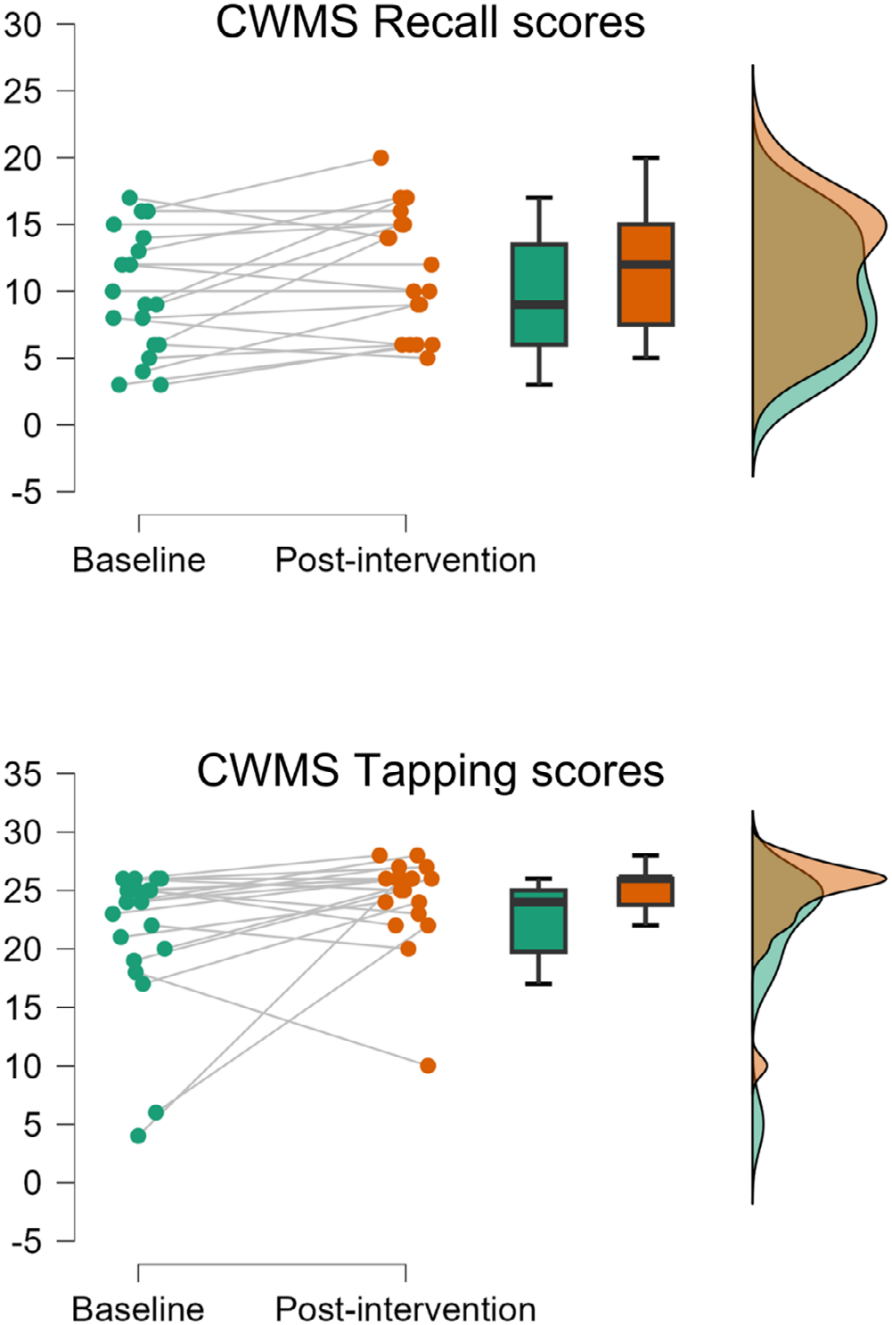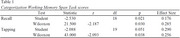# Cognitive Training and Transcranial Direct Current Stimulation in Parkinson's Disease

**DOI:** 10.1002/alz70857_103596

**Published:** 2025-12-25

**Authors:** Raphael Lopes Sartori, Marcelo J Aureliano, Vitor Tumas, Maria Paula Foss

**Affiliations:** ^1^ Faculty of Philosophy, Sciences, and Letters of the University of São Paulo, Ribeirão Preto, São Paulo, Brazil; ^2^ Clinical Hospital of the Ribeirão Preto Medical School of the University of São Paulo (HCFMRP‐USP), Ribeirão Preto, São Paulo, Brazil

## Abstract

**Background:**

Parkinson's disease (PD) often presents cognitive symptoms up to a decade before diagnosis. While cognitive training (CT) can enhance functions like working memory, further research is essential to validate its clinical applications. Combining CT with other interventions, such as physical exercise or neuromodulation, is a promising area of study. Transcranial direct current stimulation (tDCS), a non‐invasive method, has shown potential to improve cognitive functions when paired with specific tasks. Evidence suggests that tDCS may be a safe and effective strategy for addressing cognitive impairments in PD.

**Methods:**

The study included individuals aged 50–80 years diagnosed with PD and no signs of dementia. Twenty participants were randomly assigned to either an active tDCS group (*n* = 10) or a placebo‐controlled group (*n* = 10). Both groups received cognitive training combined with anodal tDCS targeting the left dorsolateral prefrontal cortex. The tDCS protocol utilized a 5 cm x 5 cm electrode delivering 2 mA for 30 minutes per session across five sessions. Neuropsychological assessments were conducted during the first and last sessions to evaluate outcomes. The primary task, the Categorization Working Memory Span Task (CWMS), measured cognitive training effects. Participants recalled target words from word lists and tapped the table upon hearing animal names, engaging working memory and attention. Data analysis employed the Wilcoxon signed‐rank test.

**Results:**

Preliminary data from 20 participants showed small but significant improvements in Recall (*p* = 0.030) and Tapping (*p* = 0.038) performance on the CWMS task after the intervention, regardless of tDCS condition. Neuropsychological assessments revealed no significant changes in mood or overall cognitive measures.

**Conclusions:**

Early findings indicate that cognitive training combined with tDCS can enhance working memory in PD patients, particularly for trained tasks. These results underscore the potential of combined interventions for mitigating cognitive deficits in PD and support the need for further research with larger sample sizes.